# Treatment of oil refinery effluent using bio-adsorbent developed from activated palm kernel shell and zeolite†

**DOI:** 10.1039/d0ra03307c

**Published:** 2020-06-24

**Authors:** Kwong Chia Jun, Abdul Aziz Abdul Raman, Archina Buthiyappan

**Affiliations:** Department of Chemical Engineering, University of Malaya 50603 Kuala Lumpur Malaysia azizraman@um.edu.my +60 3 7967 5319 +60 3 7967 5300

## Abstract

This study investigated the potential of palm kernel shell (PKS) as a biomass feed for adsorbent production. This work aims at synthesizing green adsorbent from activated PKS by integrating iron oxide and zeolite. The newly developed adsorbents, zeolite-Fe/AC and Fe/AC, were analyzed for surface area, chemical composition, magnetic properties, crystallinity, and stability. The adsorbent efficiency in removing effluent from the palm oil mill was evaluated. The influence of operating parameters, including adsorbent dosage, H_2_O_2_, reaction time, and initial solution pH for adsorption performance was studied. The Fourier transform infrared analysis revealed that the adsorbents contain functional groups including OH, N–H, C=O and C=C, which are essential for removing pollutants. The SEM-EDX analysis shows holes in the adsorbent surface and that it is smooth. The adsorption study revealed that under optimized conditions, by using 4 g L^−1^ of adsorbent and 67.7 mM H_2_O_2_, zeolite-Fe/AC was able to remove 83.1% colour and 67.2% COD within 30 min. However, Fe/AC requires 5 g L^−1^ of adsorbent and 87.7 mM to remove 86.8 percent and 65.6 percent, respectively. This study also showed that zeolite-Fe/AC has higher reusability compared to Fe/AC. Among Freundlich and Temkin models, the experimental data were found to be best fitted with the Langmuir isotherm model. The kinetic analysis revealed that for both adsorbents, the adsorption process fitted the pseudo-second-order model (*R*^2^ = 0.9724). The finding reflects monolayer adsorption of zeolite-Fe/AC and Fe/AC. This study thus demonstrates the applicability of low-cost green adsorbents produced from PKS to treat oil refinery effluent and other recalcitrant wastewaters.

## Abstract 2

This study investigated the potential of palm kernel shell (PKS) as a biomass feed for adsorbent production.

## Graphical abstract

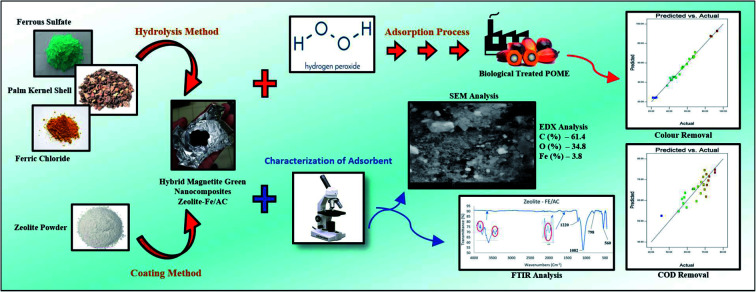

## 1 Introduction

Approximately 1500 million tons of agricultural waste is produced and disposed of annually in landfills.^1^ Researchers have recently devoted much attention to converting agricultural waste into useful products such as green adsorbents, biofuels, enzymes, vitamins, antioxidants, animal feed, antibiotics, and other chemicals.^2^ The use of agricultural waste adsorbents has several benefits over the use of conventional adsorbents, such as better biodegradability, high abundance and simple methods of collection and preparation. Livestock waste also has a better surface area of active sites, and functional groups such as hydroxyl, amino and carboxylic groups leading to high adsorption efficiency.^3^ Based on the published results, peanut shells, coconut shell,^4,5^ banana peel,^6^ palm kernel shell,^7^ garlic peel^8,9^ and rice husks^10^ can be used as an efficient and environmentally friendly bio-adsorbent for the removal of various heavy metals, anionic and cationic dyes, and persistent organic pollutants. A low-cost, readily available adsorbent should be formulated because adsorption is one of the most efficient treatment technologies for the removal of extremely recalcitrant pollutants especially from chemical and pharmaceutical production. This is because industrial wastewaters often contain substances that need to be treated before being discharged into a biological treatment plant and subsequent water bodies.

In Malaysia, palm oil production is one of the major agricultural industries, providing 37.9% of the agricultural contribution to GDP.^11^ However, every ton of fresh fruit bunches (FFB) processed produces different types of wastes such as 60 per cent of palm oil mill effluent (POME), 23 per cent of empty fruit bunches (EFB), 12 per cent of mesocarp fibers and 5 per cent palm kernel shell.^6^ Among them, POME is the largest wastes generated from the palm oil mills production with high BOD, COD, TS, TSS, colour more than 500 ADMI *etc.*^12^ POME is mainly generated from crude palm oil (CPO) production line through sterilizer condensate, sludge clarification and hydrocyclone.^13^ Approximately, 5 to 7.5 tons of POME discharge of every ton of fresh fruit bunches (FFB) processed.^12^ Since POME is considered as highly recalcitrant, the Department of Environment is started to be more stringent in standard discharge limits to control water pollution. The characteristics of the raw POME and DOE standard discharge limits has been summarized in Table 1.^12,14^

**Table:** Characteristics of raw POME and DOE standard discharge limits

Parameters	Average value	DOE discharge limit (Malaysia)
Temperature (°C)	85	45
pH	4.2	5–9
Biochemical oxygen demand, BOD (mg L^−1^)	25 000	100
Chemical oxygen demand, COD (mg L^−1^)	51 000	—
Oil & grease, O&G (mg L^−1^)	6000	50
Total solids, TS (mg L^−1^)	40 000	1500
Total suspended solids, TSS (mg L^−1^)	18 000	400
Total volatile solids, TVS (mg L^−1^)	34 000	—
Total nitrogen (mg L^−1^)	750	200
Colour (ADMI)	>500	200

Presently various technologies including advanced oxidation processes,^15^ membrane technology^16^ and chemical coagulation and flocculation,^17,18^ adsorption,^19,20^ nanofiltration^21^ have been successfully used to treat POME. Among them, processes has attracted much researcher due to its simplicity, lower operating cost and higher efficiency compared to physical and chemical techniques.^20,22^ However, one of the main challenges in the application of adsorption processes, is the selection of suitable material as the cost of operation can be significantly reduced with the usage cheaper and environmentally friendly adsorbents. So, the researchers are finding alternative adsorbents which are easily available and cheaper. Adsorbents derived from agricultural waste such as fruit peels, tea waste, fruit seeds, and bagasse consists of lignin, cellulose, potentially makes them effective adsorbent for the removal of various organic and inorganic pollutants.

On the other hand, palm kernel shell (PKS) is one of the significant waste discharging from the palm oil mill industry, producing approximately 2 million tons annually.^6,23^ PKS characteristics, such as large cavities and porosity, inexpensive, readily available and adsorption affinity, can be an excellent organic sorbents for pollutant removal.^24,25^ Past studies have confirmed that PKS could remove heavy metals, dyes, persistent organic pollutants and organic contaminants.^26,27^ Most raw PKS, however, do not have adequate adsorption efficiency, stability, good separation, and relevant use in real wastewater. Gautam and others (2013) and Zhang and others (2020) reported that various methods such as chemical modification, physical modification, biological modification, mineral impregnation, and magnetic modifications could be used to improve the efficiency of raw biomass.^28,29^ PKS composite adsorbents can also increase their adsorption performance by combining with other powerful adsorbents, inorganic compounds and organic compounds based on previous studies.^29–31^ Many materials can be used to hybridize or combined with biomass adsorbent such as iron oxide, titanium dioxide,^32^ graphene oxide,^33^ magnesium oxide,^34^ zeolite, polymer,^35^ spent shiitake substrate,^36^ sulfone^37^ and others to increase its active sites for better adsorbent performance.

Currently, iron species such as zero-valent iron Fe_3_O_4_, Fe_2_O_3_, FeS, and other iron core are extensively used for adsorbent modification for increasing the surface area and hydraulic conductivity. Magnetically modified biomass adsorbents have attracted much attention with the advantages of cost-effectiveness, environmental friendly, high reactivity, facile availability, and easily recovered due to magnetic property.^38–40^ Based on the previous studies, various types of low-cost biomass such as wheat straw,^41^ macroalgae biomass^42^ and sugarcane bagasse^43^ have been incorporated with iron oxides to remove both organic and inorganic pollutants. According to the study conducted by Hua and others (2018) incorporation of iron oxide in the biomass adsorbent able to increase the efficiency of separation process and adsorption capacity of adsorbent.^44,45^ Besides, the presence of oxidant together with iron oxide helps to initiate the Fenton reaction, which produce hydroxyl radicals and aid the adsorption process.^46^

On the other hand, zeolite is an inorganic material that is known for its ion exchange power, high porosity, large surface area, high regeneration potential, strong acidic stability, readily available and low cost.^47^ It also has a unique feature that allows other molecular dimensions to move through and shows good cations selectivity.^47,48^ Many studies also have reported that zeolite is very effective for removal of organic compound and heavy metals from wastewater.^49–53^ Shavandi and others (2012) has reported that zeolite shows great adsorption capacity particular on POME obtained from aerobic pond on removal of heavy matter.^54^ Therefore, combining zeolite and iron oxide with activated carbon palm kernel shell could work as a strong capping heterogeneous adsorbent for cationic and anionic ion in adsorption process. The hybrid adsorbent that develop also can offer a great possibility in term of the adsorption efficiency, cycle of regeneration and cost-effectiveness of adsorption process. However, so far in the literature, the application of iron oxide and zeolite for the modification of activated carbon–PKS has not been carried out yet. Therefore, the novelty of this study is the synthesis of hybrid green adsorbent and used it to treat real wastewater, with the aid of oxidant.

Therefore, the aim of this study is to develop green hybrid adsorbent from activated PKS by integrating iron oxide and zeolite. The newly developed adsorbent is used to treat, biologically treated POME through adsorption process. This study would provide a promising choice for a low cost and eco-friendly treatment of POME as well as opens a new renew to utilize by-product of palm oil processing, it is expected that the results in this study may provide some guidance for the step towards zero-discharge and sustainability in the palm oil industry.

## 2 Methodology

### 2.1 Materials

Biologically treated POME was collected from local palm oil manufacturer in Rawang Selangor Malaysia, and palm kernel shell activated carbon was purchased from Pacific Activated Carbon, Malaysia. Hydrogen peroxide 33% (H_2_O_2_), ferrous sulfate (FeSO_4_·7H_2_O), ferric chloride (FeCl_3_·6H_2_O), sodium hydroxide (NaOH), sulfuric acid (H_2_SO_4_), zeolite and ethanol have been purchased from Sigma-Aldrich (M) Sdn Bhd. All the chemicals were reagent grade and were used without further purification. Ultrapure water was used for the preparation of solutions.

### 2.2 Synthesis of Fe/AC

5 g of ground activated carbon, 3.66 g FeSO_4_·7H_2_O and 6.66 g FeCl_3_·6H_2_O were placed into a 500 mL beaker containing 100 mL of ultrapure water. The solution was stirred and heated to 65 °C for mixing propose. After 30 minutes of stirring, the solution was cooled down to 40 °C. Then the pH was adjusted to 10–11 to precipitate the iron hydroxides by using 5 M NaOH solution, and the solution was stirred for an hour. After that, the solution was left overnight and covered with cling film. The pipette was used to remove the supernatant, and then precipitates were washed with ultrapure water first then was rinsed with ethanol. After the ethanol drained, the precipitate was moved to the aluminium tray and dried at 80 °C for about 4 hours. Then, the precipitate was washed by using ultrapure water, and the magnetic rod was used to collect the activated carbon particles. The magnetically activated carbon particles dried at 80 °C overnight. After that, the dried Fe/AC was stored in an airtight bottle.

### 2.3 Synthesis of zeolite-Fe/AC

2.5 g of the zeolite and 5 g Fe/AC (0.5 : 1) were placed into a 500 mL beaker containing 100 mL of ultrapure water and was stirred for 6 hours at 60 °C. Then the mixture was placed in an ultrasonic bath for 45 min, and the mixture was left at room temperature for 24 hours. The mixture was then washed and centrifuged to separate the adsorbent from the solution. Lastly, the synthesis adsorbent (zeolite-Fe/AC) was dried in the oven at 60 °C overnight.^55^

### 2.4 Characterization of adsorbents

The surface functional groups of the synthesized adsorbent were studied using Fourier Transformation Infrared (FTIR) with a Perkin Elmer Spectrometer (Frontier) in the absorption range of 500–4000 cm^−1^. The morphology and chemical composition of the adsorbent were examined with a Scanning Emission Microscopy (SEM) (Phenom ProX)^56^ and Energy Dispersive X-ray (EDX). The changes in the morphology of the adsorbent were also analyzed using X-ray Diffraction (XRD) analysis.^57^ The magnetic properties of the adsorbents were evaluated by vibrating sample magnetometer (VSM) (LakeShore 340).

### 2.5 Adsorption study

The adsorbent capacity (zeolite-Fe/AC) was evaluated using adsorption processes with the aid of oxidants to treat POME. The initial pH of the POME was adjusted to desire condition by using 0.5 M H_2_SO_4_ acid and 1 M NaOH alkaline. The desired amount of zeolite-Fe/AC adsorbent and H_2_O_2_ were added into the sample POME solution, and the sample solution was stirred continuously at a constant rate of 200 rpm. At the end of each experiment, the sample solution was obtained and filtered to remove the adsorbent. Thus, colour removal was measured immediately. Right after the colour removal reading was taken, the pH of the sample solution was adjusted to the alkaline base by using 1 M NaOH with the ratio of every 1 mL of 200 mM of H_2_O_2_ solution with 10 mL of 1 M NaOH. For the reusability study, the used adsorbent was washed with ultrapure water, followed by 0.1 M NaOH for 10 minutes. After that, the used adsorbent was washed with distilled water thrice. Lastly, the adsorbent was dried in an oven for 1.5 hours and stored for the next experiment. Yet, the performance of the Fe/AC also evaluated through the same procedure as evaluating the performance of zeolite-Fe/AC. All the experiments were repeated twice.

### 2.6 Experimental design and data collection

Design-Expert Software (Version 10) was used to design the experiments. Response Surface Methodology (RSM) – Central Composite Design (CCD) was used in this study to optimize four independent variables: the dosage of adsorbent, dosage of H_2_O_2_, reaction time and pH value. Preliminary experiments were carried to determine the ranges for the operating parameters. The adsorption study was conducted to identify the optimum operating conditions used including dosage of adsorbent 1–5 g L^−1^, the dosage of H_2_O_2_ 40–200 mM, reaction time 10 min to 60 min and pH value from 3 to 9. Analysis of variance (ANOVA) was used to evaluate the interaction between independent variables and the responses. The quality of the fit regression model was expressed by the determination coefficient, *R*^2^. In terms of statistical significance, Fishers *F*-test was used. It can determine whether the model was accepted or rejected based on the probability (*p*-value) with a 95% confidence level.

### 2.7 Data analysis

UV-Vis spectrophotometer was used to determine the COD and colour removal, according to the American Public Health Association (APHA) standard method. The decolourization efficiency and COD removal have been calculated using eqn (1) and (2).1
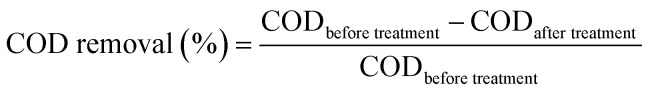
2
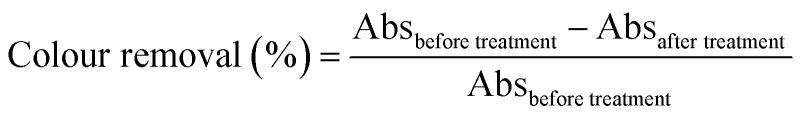


## 3 Results and discussion

### 3.1 Characterisation of adsorbents

#### (a) FTIR analysis

**Fig. 1:**
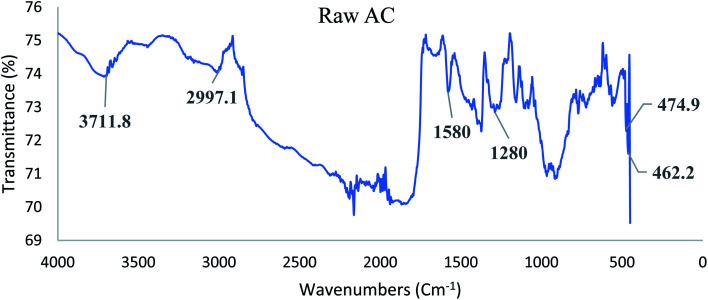
FTIR spectrum of raw palm kernel shell activated carbon (AC).

**Fig. 2:**
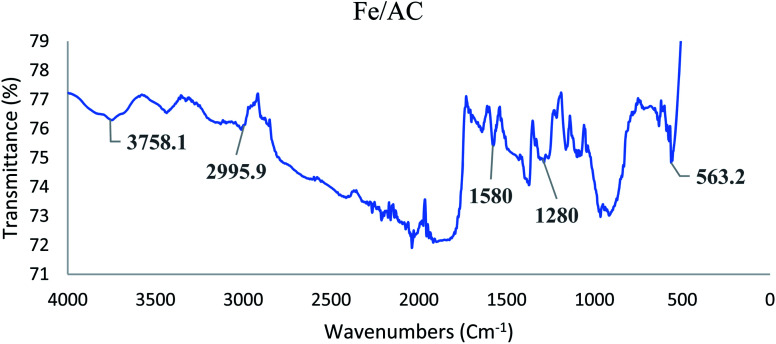
FTIR spectrum of modified palm kernel shell activated carbon (Fe/AC).

Fig. 1 and 2 display the FTIR spectra for raw palm kernel shell activated carbon (AC) and modified palm kernel shell activated carbon (Fe/AC). AC and Fe/AC reported a sharp peak at 3711 cm^−1^ and 3000 cm^−1^, respectively suggesting the O–H a stretching. The sharp peak at 2996 cm^−1^ found in both AC and Fe/AC shows the presence of asymmetric and symmetric C–H stretching.^58,59^ Besides, C=C stretching represented by the peak at 1580 cm^−1^ in both AC and Fe/AC adsorbents. While the peak at 1280 cm^−1^ s indicates the presence of C=O stretching.^60^ The presence of C=O is probably due to the presence of the amide group in the protein originated from the palm kernel shell. The peak at 560 cm^−1^ which is only seen in Fe/AC confirmed the presence of iron oxide.^61^ The FTIR analysis results of the raw palm kernel shell AC obtained in this study is similar to the results reported by Misnon and others (2015).^60^

**Fig. 3:**
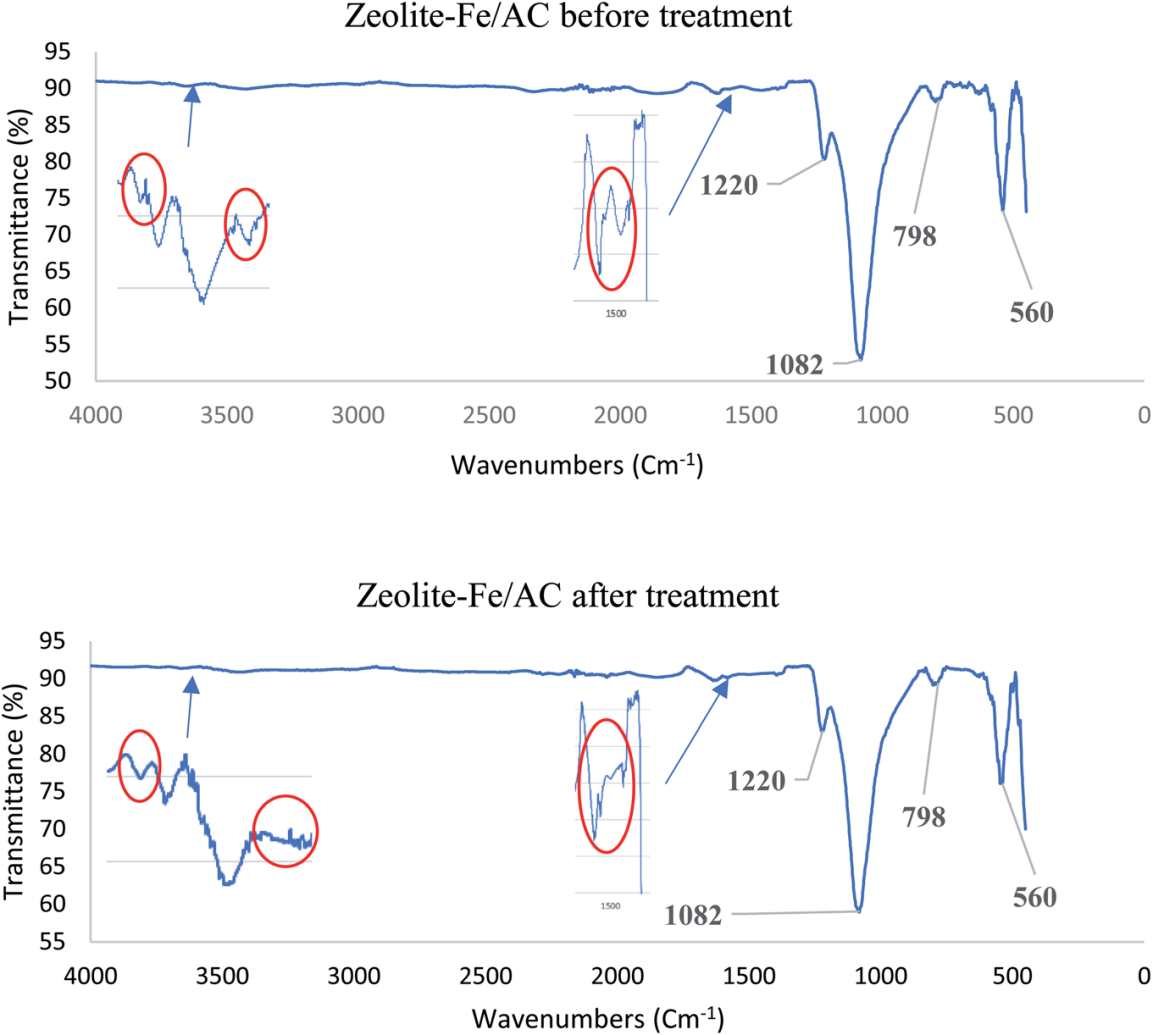
FTIR spectrum of zeolite-Fe/AC (a) before treatment (b) after treatment.

Fig. 3(a) and (b) shows the FTIR spectra for zeolite-Fe/AC before and after treatment. The presence of a peak at 1082 and 798 cm^−1^ indicates the asymmetric and symmetric stretching vibrations which corresponding to SiO_4_ or AlO_4_ structure of zeolite according to few articles.^62,63^ Also, peak at around 560 cm^−1^ in zeolite-Fe/AC attributes to Fe–O bond stretching, and it confirmed the presence of iron oxide.^61^ The peak at 1220 cm^−1^ ascribed to the presence of epoxy stretching. The stretching of hydroxyl groups of the zeolitic structure was represented by the broad peak from 3700–3400 cm^−1^.^62^ By comparing the spectra of the zeolite-Fe/AC before and after adsorption, it could be seen that some of the peaks have been eliminated or moved. The reduced peak in the region from 3400 cm^−1^ to 3700 cm^−1^ and 1508 cm^−1^ represent the OH stretching and N–H deformation, respectively. This proved that the adsorption process involved OH and NH functional groups.^63^

#### (b) Scanning Emission Microscopy (SEM)

The raw AC displayed a rough, irregular, and amorphous structure as can be seen in S1(a).† However, the morphology of modified activated carbon (Fe/AC) in S1(b)† exhibited a crystal surface with smaller pore sizes. Patches of white crystals may indicate the presence of iron oxide nanoparticles.^64^ The morphology of zeolite-Fe/AC in S1(c)† shows porous, loose texture and presence of crystal surface with reduced, which confirmed the presence of zeolite particles.^62^ The result obtained in line with the study reported by Cheng and others (2016).^65^

#### (c) Energy Dispersive X-ray (EDX)

The chemical compositions of AC, Fe/AC and zeolite-Fe/AC were examined, and the results of the EDX analysis is shown in Tables 2 and S2.† PKS–AC, comprised of 60.7% of C, 24.6% of O. While, Fe/AC consists of lesser carbon (40.9%) and higher oxygen (49.9%) and Fe (9.1%). The reduction of C composition most likely due to the introduction of iron oxide in raw AC. On the other hand, the zeolite-Fe/AC shows the decrease of Fe and O compositions due to the presence of zeolite participles. Zeolite is known as carbon material, so there is an increase in the content of C in zeolite-Fe/AC.^58,66^

**Table:** Chemical composition of AC and Fe/AC

Material	C (%)	O (%)	Fe (%)	Ca (%)
AC	60.7	24.6	—	14.6
Fe/AC	40.9	49.9	9.1	—
Zeolite-Fe/AC	61.4	34.8	3.8	—

#### (d) X-ray Diffraction (XRD) analysis

**Fig. 4:**
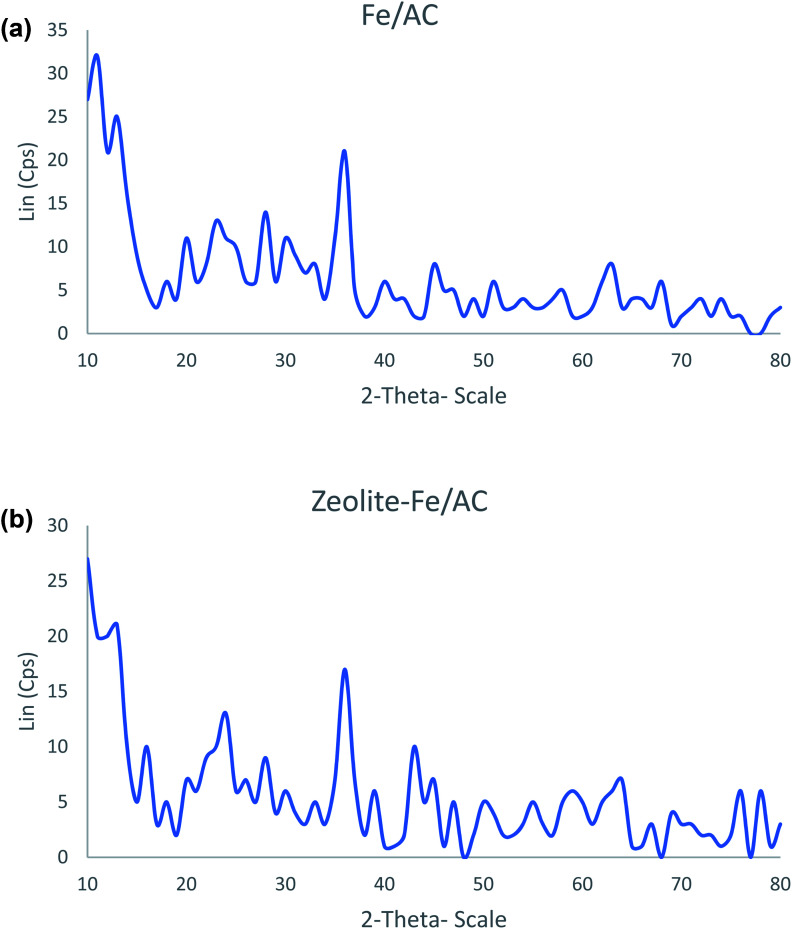
The XRD spectra of (a) Fe/AC and (b) zeolite-Fe/AC.

Fig. 4 shows the X-ray diffraction (XRD) analyses of both Fe/AC and zeolite-Fe/AC. The iron peaks are identified at *θ*: 30.2, 32.1, 35.5, 43.3, 53.5, 56.8, 62.7 and 73.9. This result further supports the SEM/EDX analysis that the iron magnetic nanoparticles have been successfully coated and incorporated into PKS. Fig. 4(b) also shows that zeolite-Fe/AC contains lesser iron oxide compared to Fe/AC as zeolite particles have replaced it. Similar results have also reported by Jianhua Qu and others (2020).^67^

#### (e) Vibrating sample magnetometer (VSM)

S3† shows that Fe/AC has the saturation magnetization value of about 16.6 emu g^−1^. Whereas the zeolite-Fe/AC only has a saturation magnetization value of 10.6 emu g^−1^. Even though zeolite-Fe/AC has lower magnetic properties, it meets the minimum criteria of solid–liquid separation.^68^ The result obtained in line with the study of Javadian and others (2020).^69^

### 3.2 Adsorption study

#### (a) Experimental design

Tables 3 and 4 summarized the experimental design suggested by RSM. The result clearly shows that both adsorbents managed to remove more than 80% of colour and 70% of COD.

**Table:** Experimental design suggested by RSM, and the result obtained on colour removal

Run	Dosage of H_2_O_2_ (mM)	Independent variables			Colour removal efficiency (%)	
		Adsorbent (g L^−1^)	Reaction time (min)	pH	Fe/AC	Zeolite-Fe/AC
1	120	3	35	6	76.5	43.9
2	40	1	60	9	69.1	24.1
3	200	1	60	9	69.4	42.3
4	40	5	10	3	83.7	86.1
5	120	3	15	6	78.3	43.1
6	200	5	60	9	79.1	55.5
7	40	5	60	9	79.9	40.8
8	200	1	10	9	70.0	41.7
9	280	3	35	6	73.9	54.1
10	120	3	35	6	77.9	44.9
11	40	1	60	3	76.5	62.4
12	200	5	10	3	82.7	87.1
13	40	1	10	9	71.4	21.7
14	120	3	35	12	74.2	67.8
15	120	3	35	6	76.1	45.3
16	120	3	35	6	76.1	44.5
17	200	1	60	3	73.0	68.8
18	200	1	10	3	77.1	66.8
19	120	7	35	6	82.7	66.6
20	200	5	60	3	80.1	93.4
21	120	3	35	6	74.6	46.1
22	40	1	10	3	79.5	59.6
23	200	5	10	9	80.1	55.1
24	40	5	10	9	80.3	47.7
25	120	1	35	6	70.0	30.4
26	40	5	60	3	76.5	93.4
27	40	3	35	6	78.5	25.7
28	120	3	35	0	—	—
29	120	3	35	6	76.7	46.5
30	120	3	85	6	76.1	51.5

**Table:** Experimental design suggested by RSM, and the result obtained on COD removal

Run	Dosage of H_2_O_2_ (mM)	Independent variables			COD removal efficiency (%)	
		Adsorbent (g L^−1^)	Reaction time (min)	pH	Fe/AC	Zeolite-Fe/AC
1	120	3	35	6	74.7	63.7
2	40	1	60	9	65.0	55.3
3	200	1	60	9	73.7	67.9
4	40	5	10	3	73.0	71.1
5	120	3	15	6	73.2	67.6
6	200	5	60	9	76.3	75.0
7	40	5	60	9	71.0	57.1
8	200	1	10	9	72.9	66.1
9	280	3	35	6	80.0	69.7
10	120	3	35	6	74.7	63.7
11	40	1	60	3	71.0	58.9
12	200	5	10	3	80.8	68.9
13	40	1	10	9	67.0	45.8
14	120	3	35	12	70.0	70.5
15	120	3	35	6	74.5	63.9
16	120	3	35	6	75.0	63.4
17	200	1	60	3	75.8	69.2
18	200	1	10	3	77.6	69.7
19	120	7	35	6	75.5	72.4
20	200	5	60	3	79.7	75.5
21	120	3	35	6	74.7	63.7
22	40	1	10	3	71.0	58.4
23	200	5	10	9	75.3	70.8
24	40	5	10	9	69.0	57.9
25	120	1	35	6	73.4	65.3
26	40	5	60	3	75.0	71.8
27	40	3	35	6	67.9	59.7
28	120	3	35	0	—	—
29	120	3	35	6	74.7	63.7
30	120	3	85	6	74.7	69.2

### 3.3 Statistical analysis

#### (a) ANOVA – CCD for colour and COD removal

Tables 5 and 6 shows the analysis of variance (ANOVA) for colour and COD removals for both Fe/AC and zeolite-Fe/AC. Reduced 2FI model and reduced quadratic model were developed for Fe/AC and zeolite-Fe/AC, respectively for the colour removal efficiency. Whereas, the linear model and reduced 2FI were suggested by RSM-CCD for Fe/AC and zeolite-Fe/AC respectively for the COD removal. The final equations of colour and COD removal in terms of coded factors were expressed by eqn (3) and (4), (5) and (6). The positive sign of the terms in the equation implies the synergistic effect while the negative sign suggests antagonistic effect.

**Table:** Analysis of variance (ANOVA) for colour removal efficiency^a^

Source	Sum of squares	df	Mean square	*F*-Value	*p*-Value
**Fe/AC**					
Model	425.8	7	60.8	45.4	<0.0001
A – dosage of H_2_O_2_	8.8	1	8.8	6.5	0.0184
B – dosage of adsorbent	283.1	1	283.1	211.5	<0.0001
C – pH	60.3	1	60.3	45.0	<0.0001
D – reaction time	27.2	1	27.2	20.3	0.0002
AB	4.7	1	4.7	3.5	0.0763
BC	32.1	1	32.1	24.0	<0.0001
CD	10.0	1	10.0	7.4	0.0126
Lack of fit	22.4	16	1.4	1.2	0.4462
*R*-squared	0.938				
Adj *R*-squared	0.917				
Pred *R*-squared	0.871				
Adeq. precision	24.6				
Eqn (3)	76.7–0.6A + 3.4B − 1.7C − 1.2D + 0.5AB + 1.4BC + 0.8CD				

**Zeolite-Fe/AC**					
Model	10 098.0	9	1122.0	322.2	<0.0001
A – dosage of H_2_O_2_	481.9	1	481.9	138.4	<0.0001
B – dosage of adsorbent	2676.5	1	2676.5	768.6	<0.0001
C – pH	5557.0	1	5557.0	1595.9	<0.0001
D – reaction time	42.0	1	42.0	12.1	0.0025
AB	51.7	1	51.7	14.8	0.0011
AC	130.8	1	130.8	37.6	<0.0001
BC	68.7	1	68.7	19.7	0.0003
CD	29.8	1	29.8	8.6	0.0086
C^2	3188.5	1	3188.5	915.7	<0.0001
Lack of fit	61.4	14	4.4	4.6	0.0511
*R*-squared	0.993				
Adj *R*-squared	0.990				
Pred *R*-Squared	0.980				
Adeq. precision	62.8				
Eqn (4)	45.4 + 4.5A + 10.6B − 17.8C + 1.3D − 1.8AB + 2.9AC − 2.1BC − 1.4CD				

a A: dosage of H_2_O_2_, B: dosage of adsorbent, C: pH value, D: reaction time.

**Table:** Analysis of variance (ANOVA) for COD removal efficiency^a^

Source	Sum of squares	df	Mean square	*F*-Value	*p*-Value
**Fe/AC**					
Model	354.5	4	88.6	77.9	<0.0001
A – dosage of H_2_O_2_	230.1	1	230.1	202.3	<0.0001
B – dosage of adsorbent	38.4	1	38.4	33.7	<0.0001
C– pH	85.4	1	85.4	75.1	<0.0001
D – reaction time	0.5	1	0.5	0.5	0.5017
Lack of fit	27.2	19	1.4	50.9	0.0002
*R*-squared	0.928				
Adj *R*-squared	0.916				
Pred *R*-squared	0.892				
Adeq. precision	29.8				
Eqn (5)	73.8 + 3.1A + 1.3B − 2.1C + 0.2D				

**Zeolite-Fe/AC**					
Model	896.4	5	179.3	12.5	<0.0001
A – dosage of H_2_O_2_	480.4	1	480.4	33.5	<0.0001
B – dosage of adsorbent	213.6	1	213.6	14.9	0.0008
C – pH	69.4	1	69.4	4.8	0.0382
D – reaction time	27.7	1	27.7	1.9	0.1776
AC	105.3	1	105.3	7.3	0.0125
Lack of fit	329.7	18	18.3	651.8	<0.0001
*R*-squared	0.731				
Adj *R*-squared	0.673				
Pred *R*-Squared	0.542				
Adeq. precision	12.9				
Eqn (6)	65.5 + 4.5A + 2.9B − 1.9C + 1.1D + 2.6AC				

a A: dosage of H_2_O_2_, B: dosage of adsorbent, C: pH value, D: reaction time.

Tables 5 and 6 present the result of statistical analysis for both adsorbents. The *F*-values shows that model of both adsorbents was significant at 95% confidence level. For Fe/AC and zeolite-Fe/AC, the *F*-values were 45.4 and 322.2 respectively for colour removal. While *F*-values for COD removal for Fe/AC and zeolite-Fe/AC were 77.9 and 12.5. The *p*-values <0.0500 for A, B, C, D, BC and CD for Fe/AC indicates that the parameters are significant for colour removal. Whereas for zeolite-Fe/AC, A, B, C, D, AB, AC, and CD are significant. On the other hands, for COD removal, A, B and C parameters are significant for Fe/AC, and A, B, C and AC for zeolite-Fe/AC.

The *F*-value should be insignificant to ensure that the model is fit. The result shows that both models for colour removal are insignificant. In terms of *R*^2^ for Fe/AC, colour and COD removal reported having 0.938 and 0.928, respectively. Whereas, zeolite-Fe/AC has the *R*^2^ of 0.994 and 0.731 for colour and COD removal, respectively. Both adsorbents reported to have *R*^2^ close to 1, and the differences between adjusted and predicted *R*^2^ were <0.5, which indicated that the high accuracy of the model.^70^ Adequate precision compares the range of the predicted values at the design points to the average prediction error. As can be seen in Table 5, both the model for colour removal shows the adequate precision ratios of 24.6 (Fe/AC) and 62.8 (zeolite-Fe/AC). And adequate precision ratios for COD removal of Fe/AC and zeolite-Fe/AC were 29.8 and 12.9, respectively. A ratio greater than 4 indicates that models are sufficient to navigate the CCD-model. S4(a), (b), S5(a) and (b)† shows the graph of predicted *versus* actual for Fe/AC and zeolite-Fe/AC.

### 3.4 Optimization and model validation

Table 7 shows the predicted and the experimental results obtained for selected optimum conditions. The experimental values are close enough to the predicted values, with a deviation of less than 5%; therefore, the model is considered reliable for this study. As can be seen in Table 7, Fe/AC had the highest colour removal (86.8%) compared to zeolite-Fe/AC (83.1%). However, zeolite-Fe/AC achieved higher COD removal efficiency of 67.2% compared to Fe/AC.

Optimized conditions

**Table:** 

Adsorbents	Dosage of adsorbent (g L^−1^)	Dosage of H_2_O_2_ (mM)	Reaction time (min)	pH value
Fe/AC	5	84.0	30	3
Zeolite-Fe/AC	4	67.7	30	3

**Table:** 

Adsorbents	Colour removal efficiency (%)		COD removal efficiency (%)	
	Predicted	Experimental	Predicted	Experimental
Fe/AC	82.7	86.8	—	63.6
Zeolite-Fe/AC	81	83.1	—	67.2

### 3.5 Kinetic study

Results of kinetic study for Fe/AC and zeolite-Fe/AC under the optimum condition on colour removal has been presented in Table 8. It shows that the adsorption process happened rapidly within 10 to 30 min in experiment.^71,72^ There is an only slight increase (<1%) after 6 minutes of the kinetic study experiment as recorded. In the present study, the colour removal efficiency was tested on zero-, first- and second-order reaction kinetic. The kinetics study for zeolite-Fe/AC and Fe/AC with the aid of oxidants shows in S6.† It was found that the regression coefficients, *R*^2^ of the second-order reaction kinetic S6(c)† of zeolite-Fe/AC and Fe/AC were 0.9331 and 0.9724 respectively, which are obviously much higher than zero-order (zeolite-Fe/AC, *R*^2^ = 0.9211 and Fe/AC, *R*^2^ = 0.9412) and first-order (zeolite-Fe/AC, *R*^2^ = 0.928 and Fe/AC, *R*^2^ = 0.9632) reaction kinetic. By comparing the regression coefficients obtained, it can be concluded that the second-order reaction kinetic fit the reaction best. The results from this study were consistent with the work from ^73–75^

**Table:** Kinetic study of Fe/AC and zeolite-Fe/AC

Time (min)	Kinetic study under optimum condition on colour removal (%)	
	Fe/AC	Zeolite-Fe/AC
2	78.9	80.5
4	82.6	80.6
6	83.6	83.3
8	84.9	83.7
10	86.7	84.9

### 3.6 Adsorption isotherm study

Adsorption isotherms explained about the amount of adsorbate adsorbed by unit mass of adsorbent from the liquid phase. It is essential to carry out the analysis of adsorption equilibrium data for design optimization of an adsorption system. Adsorption isotherm shows the relationship between adsorbate adsorbed onto active site of adsorbent and the concentration of the solution.^76^ To date, various isotherms model has been used widely to analyze the adsorption data. In this study, Langmuir, Freundlich and Temkin models have been used to study the interaction of concentration of POME with adsorbent, Fe/AC and zeolite-Fe/AC.

#### (a) Langmuir adsorption isotherm

The Langmuir isotherm is referred to the monolayer adsorption of adsorbate on the adsorbent, Fe/AC and zeolite-Fe/AC. The linearized form of equation of Langmuir isotherm is as follow:^77^7
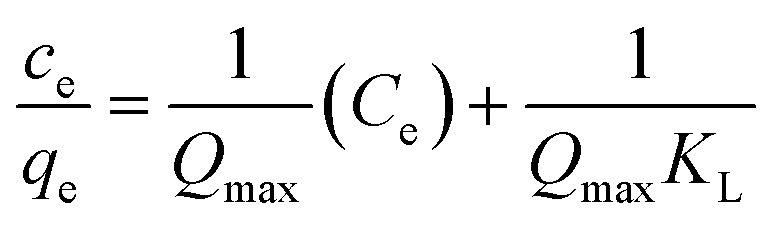


#### (b) Freundlich adsorption isotherm

Freundlich isotherm suggest multilayer adsorption on the heterogeneous surface; it also explains that the adsorption increase with increase in the concentration. The linear equation of Freundlich adsorption isotherm is shown in eqn (8):^78^

8 log *q*_e_ = (1/*n*)log *C*_e_ + log *K*_F_

#### (c) Temkin adsorption isotherm

The Temkin isotherm equation assumes that the heat of adsorption of all the molecules in the layer decreases linearly with coverage due to adsorbent–adsorbate interactions, and that the adsorption is characterized by a uniform distribution of the binding energies, up to some maximum binding energy. The linearized Temkin isotherm is given in eqn (9):

9 *q* = *B* ln *KT* + *B* ln *C*

where, *B* = *RT*/*b*. *KT* is the equilibrium binding constant (L mg^−1^) corresponding to the maximum binding energy and *b* is a constant related to the heat of adsorption. *R* is the gas constant (8.314 J mol^−1^ K^−1^) and *T* is the absolute temperature (K). A plot of *q versus* ln *C* enables the determination of the isotherm constants *KT* and *b* from the intercept and slope respectively.

Temkin isotherm explained the interaction between adsorbent–adsorbate, and it also assumes that the heat of adsorption decreases linearly with coverage. The linearized Temkin isotherm is given in eqn (10)^79^

10 *q*_e_ = *B*_1_ ln *C*_e_ + *B*_1_ ln *K*_T_

For all isotherm model above, *C*_e_ is the concentration of adsorbate adsorbed at equilibrium; *q*_e_ is the adsorption capacity of adsorbent at equilibrium concentration; *Q*_max_ is the maximum adsorption capacity; *K*_L_, *K*_f_ and *K*_T_ are the constant in Langmuir, Freundlich Temkin isotherm of adsorption respectively; 1/*n* is the adsorption intensity; *B*_1_ = *RT*/*b*; *b* is a constant related to the heat of adsorption; *R* is the gas constant (8.314 J mol^−1^ K^−1^) and *T* is the absolute temperature (K).

For Langmuir isotherm, the plot of *C*_e_/*q*_e_ against *C*_e_ will gives straight line. The Langmuir constants will be calculated form the linear plot. For Freundlich isotherm, the value of *K*_f_ and 1/*n* were obtained from the intercept and slope of linear plot in the plot of log *q*_e_ against log *C*_e_ respectively. The value of *n* varies with the heterogeneity of adsorbent. The value of *n* must be less than 10^8^. When the value 1/*n* is closer to 0, it means that the adsorbent is becoming more heterogeneous.^80^ For Temkin isotherm, value of *K*_T_ and *B*_1_ able to obtain through the plot of *q*_e_ against ln *C*_e_. All the calculated value of constant was stated in Table 9.

**Table:** Isotherm constant parameter and correlation coefficients calculated for adsorption study

Isotherm	Parameters	Adsorbent	
		Fe/AC	Zeolite-Fe/AC
Langmuir	*Q*_max_	19.2	24.1
	*K*_L_	1.2	0.36
	*R* ^2^	0.9987	0.9924
	*R*_L_	0.01	0.03
Freundlich	1/*n*	0.2	0.3
	*K*_F_	11.8	10.4
	*R* ^2^	0.9508	0.9841
Temkin	*B*_1_	2.7	4.4
	*K*_T_	69.6	7.1
	*R* ^2^	0.9709	0.9766

Based on the result summarized in the Tables 9 and S7,† the Langmuir isotherm model is fitted the best for both adsorbent, Fe/AC and zeolite-Fe/AC with the highest value of *R*^2^ of 0.9987 and 0.9924, respectively. This result proves that the formation of a monolayer adsorption at the surface of the PKS adsorbent.^79^ Furthermore, it also indicate that the active sites were homogeneously distributed on the adsorbents surface.^81^ The value of *R*_L_ obtained in this study are between 0 to 1 for both adsorbents, thus it further explained that Langmuir isotherm is a favorable model to explain the adsorption of POME.^82^ Besides, the maximum adsorption capacity *Q*_max_ of zeolite-Fe/AC (24.1) is higher than Fe/AC (19.2). This shows that the present of zeolite able to increase the surface area of the adsorbent as more amount of an adsorbate loadable on zeolite-Fe/AC. This could be explained as the specific surface area of the adsorbent and affinity of the adsorbent can be expressed by the adsorption isotherm study and maximum adsorption capacity.^83,84^ Many other published research also reported that Langmuir isotherm is more favorable for COD and color removal by using bio adsorbent^6,9,85–88^

### 3.7 Plausible adsorption mechanism

**Fig. 5:**
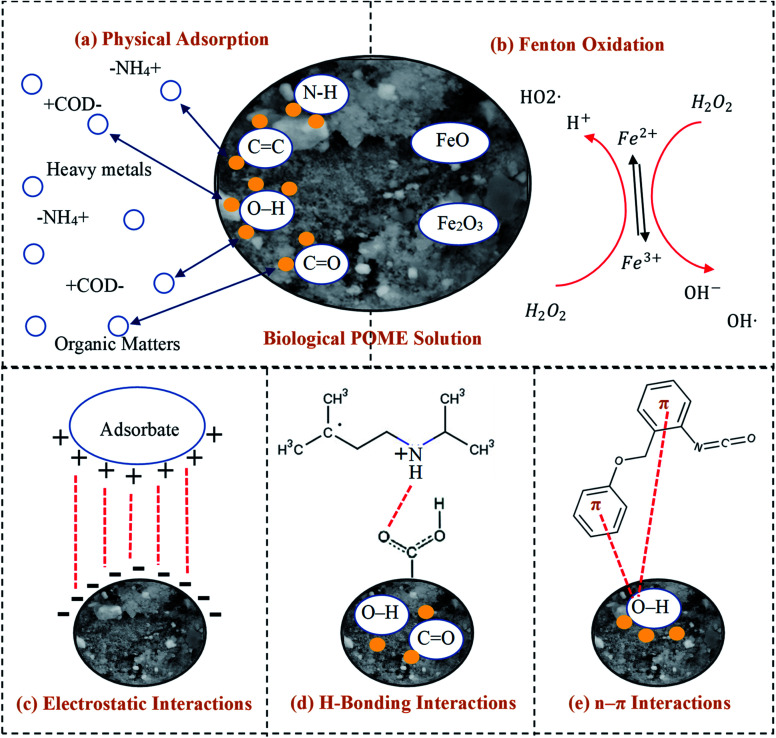
Proposed adsorption mechanisms of adsorption process with the aids of oxidants: (a) physical adsorption, (b) Fenton oxidation, (c) electrostatic interactions, (d) H-bonding interactions and (e) *n*–π interactions.

Fig. 5 presents the plausible adsorption mechanisms with the aids of oxidants for zeolite-Fe/AC adsorbent for biological POME by considering initial solution pH, as well as FTIR analysis. In this study, the pH of the POME was found to be acidic conditions (pH 3). The palm oil mill effluent (POME) contains high chemical oxygen demand (COD) and biological oxygen demand (BOD), oil and grease, suspended solids, ammonia–nitrogen, heavy metal concentration and high content of degradable organic matter.^89–91^ The adsorption behavior of pollutants present in the POME and zeolite-Fe/AC adsorbent is summarized in Fig. 5. Besides, the previously discussed FTIR analysis also clearly explain the deformation and stretching vibrations of the functional group after the adsorption of pollutants. This further describe that the possible mechanism of pollutants adsorption onto zeolite-Fe/AC could be through electrostatic interaction, hydrogen bonding and *n*–π interactions between the functional group of POME and surface of zeolite-Fe/AC adsorbent.^92,93^ The possible bond formation mechanism between organic matters and heavy metals with functional group of zeolite-Fe/AC adsorbent has shown in Fig. 5(c)–(e). The proposed mechanism that reported is in line with works reported by previous researchers, where adsorption mechanism could be through three different processes.^87,94^

Fig. 5(a) and (b) shows that there might be physical adsorption and Fenton oxidation happen simultaneously in this study. The present of iron oxide in the adsorbent will react with the oxidants (H_2_O_2_) in the reaction and it will speed up the whole reaction of adsorption process. This could be supported by the removal rate (>80%) within a short reaction time (4 min). Wei and others (2020) also reported that adsorption and oxidation can happened simultaneous with the present of oxidants.^95^ The present of iron oxide and oxidants can greatly improve the reaction time in the reaction.^96,97^

### 3.8 Reusability of adsorbents

The stability study of the developed adsorbent was evaluated over five cycles. The ability of an adsorbent to be used many times without losing its adsorbent capacity is an important consideration in industrial application.^98^ S8† and Table 10 depicts the colour removal by using the Fe/AC and zeolite-Fe/AC adsorbents over five consecutive cycles. As shown in the results, for Fe/AC, the colour removal efficiency recorded at 80.5% from 87.1% after the 5^th^ cycle. While for zeolite-Fe/AC only shows a slight decrease from 83.9% to 81.0%. Zeolite-Fe/AC was able to retain its adsorbent capability over five cycles with a minimal reduction of 2.9%, but Fe/AC had significantly impaired on its adsorbent capacity of 6.6% decrease which corresponds to the study by Pham, Lee and Kim (2016).^99^ The reason of zeolite-Fe/AC able to maintain most of its adsorbent capability probably due to the addition of zeolite particles had increased its stability of the adsorbent or due to the characteristic of zeolite that had been reported in few articles.^62,63,100,101^ Zeolite is made up of aluminium, oxygen, and metals like titanium, Tin, Zinc, *etc.* Hence it had been proven to have a special characteristic where it is able to permit the passage of molecules that below a certain size.^48^

**Table:** Reusability of Fe/AC and zeolite-Fe/AC

Adsorbent (%)	Fe/AC	Zeolite-Fe/AC
Regeneration 1st cycle	87.1	83.9
Regeneration 5th cycle	80.5	81.0
Total amount lost	6.6	2.9

### 3.9 Comparison between Fe/AC and zeolite-Fe/AC adsorbents

Table 11 shows the comparison of Fe/AC and zeolite-Fe/AC adsorbents. The result shows that both adsorbents able to achieve >80% colour removal and >65% COD removal within 30 minutes. As can be seen in Table 11, Fe/AC managed to achieve higher colour removal (86.8%) compared to zeolite-Fe/AC (83.1%). However, Fe/AC reported having lower COD removal (65.6%) in comparison to zeolite-Fe/AC (67.2%). On the other hand, zeolite-Fe/AC used lesser adsorbent and H_2_O_2_ (4 g L^−1^; 67.7 mM) compared to Fe/AC (5 g L^−1^; 84.0 mM). As can be seen in Table 10, zeolite-Fe/AC was able to retain its adsorbent capability over five cycles, with only 2.9% loss compared to Fe/AC. One of the biggest challenges of adsorption is the cost of the adsorbent and regeneration ability of conventional activated carbon.^62,98^ Therefore the minimal consumption of adsorbent and oxidants, a shorter reaction time of 30 min and reusability capacity show that both zeolite-Fe/AC and Fe/AC developed from palm kernel shell (PKS) is a great alternative of an adsorbent for adsorption process compared to conventional activated carbon. In addition, the presence of oxidant in the process usually creates a lower hydroxyl radical requirement, as a reaction such as Fenton oxidation can occur with the adsorption cycle. As explained by eqn (11) and (12), H_2_O_2_ will react with Fe^2+^ that present in the adsorbent to form OH˙ radical and Fe^3+^ will react with H_2_O_2_ to regenerate Fe^2+^ with HO_2_ and H^+^.^102^ Based on the studies of Huling (2017) and Y.-T. Chung (2017), it had been proven that oxidants were able to improve adsorption process.^103,104^

11 Fe^2+^ + H_2_O_2_ → OH˙ + Fe^3+^ + OH^−^

12 Fe^3+^ + H_2_O_2_ → HO_2_˙ + Fe^2+^ + H^+^

**Table:** Comparison of Fe/AC and zeolite-Fe/AC adsorbent

Adsorbent	pH	Dosage adsorbent (g L^−1^)	Dosage H_2_O_2_ (mM)	Reaction time (min)	Colour removal (%)	COD removal (%)
Fe/AC	3	5	84.0	30	86.8	65.6
Zeolite-Fe/AC	3	4	67.7	30	83.1	67.2

Ultimately, zeolite-Fe/AC is preferred economically over Fe/AC as less adsorbent and oxidants are needed and the better adsorption capacity. The effect of the operating parameter on the adsorption of zeolite-Fe/AC cycle is discussed in the following section.

### 3.10 Effect of operational parameters

#### (a) Effect of pH

**Fig. 6:**
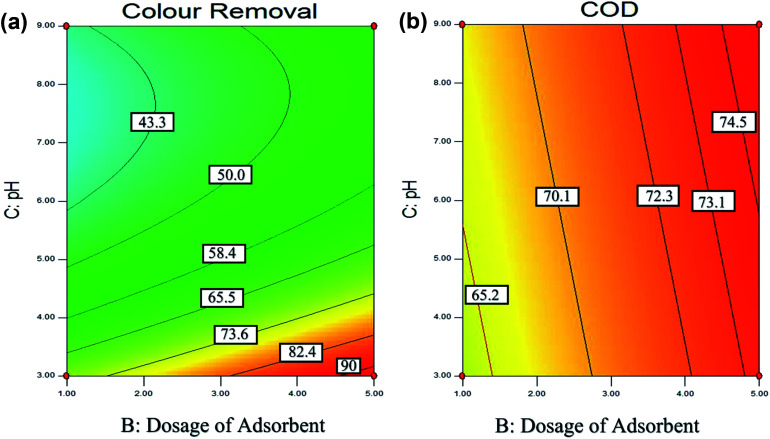
Contour (2D) plot for (a) colour (b) COD removal of pH and dosage of adsorbent.

The solution pH is an essential parameter in the adsorption process.^72^ In this experiment, the effect of pH was investigated by varying the pH between 3 to 9. Fig. 6(a) and (b) shows the effect of pH and dosage of adsorbent on the colour and COD removal, respectively. As, when the pH of the solution increase, the surface of adsorbent become more negatively charged and thus increase in the repulsion between the adsorbate and adsorbent. Therefore, this cause the removal efficiency decreases with an increase in pH.^105^ Hence, it is proven that the adsorption process for POME favors acidic condition in this study.

Fig. 6(a) shows that higher colour removal efficiency (>90%) is achieved at acidic condition, around pH 3. However, when the pH value exceeded 4, it clearly shows that there is a significant decrease in the colour removal efficiency (<65%). Besides, Fig. 6 also shows that the interaction between solution pH and dosage of the adsorbent. It shows that higher colour removal rate (>90%) and COD removal rate (>73%) achieved when the adsorbent is 5 g L^−1^ and pH 3, however lower colour removal rate (<40%), and COD removal rate (<65%) were observed at the dosage of adsorbent is 1 g L^−1^ with pH 9.

#### (b) Effect of adsorbent dosage

**Fig. 7:**
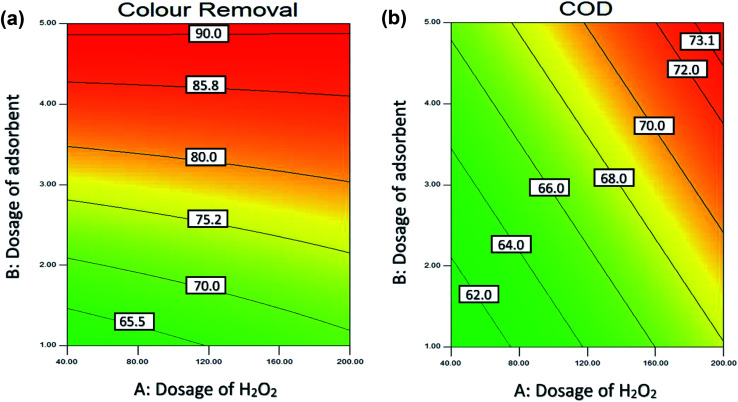
Contour (2D) plot for (a) colour (b) COD removal of adsorbent dosage and H_2_O_2_ dosage.

By varying the adsorbent dosage between 1 to 5 g L^−1^, the effect of adsorbent dosage on the colour removal was studied. Fig. 7(a) and (b) show the effective dosage of adsorbent and H_2_O_2_ dosage on the colour removal and COD removal of POME. At lower adsorbent dosage, the colour and COD removal efficiency was small as there was insufficient surface area of the adsorbent for the effective decomposition of H_2_O_2_. The dosage of adsorbent able to affect the adsorption in terms of availability of surface area for adsorption process as it allows adsorbate to penetrate the adsorption sites easily.^6^ In conclusion, the dosage of adsorbent will affect the colour removal rate significantly, and it also acts as one of the main characteristics of industrial application.^98^ A similar result was reported by Mohammed and Chong (2014).^6^

#### (c) Effect of dosage of H_2_O_2_

Fig. 7(a) and (b) shows the effect of adsorbent dosage and H_2_O_2_ dosage on the colour removal and COD removal of POME. The dosage of H_2_O_2_ act as an essential parameter in COD removal as the production of the hydroxyl radical depends on the oxidants. The previous studies shows that the presence of oxidants such as H_2_O_2_ able to increase the rate of adsorption in reaction.^103,104^ However, the excessive dosage of H_2_O_2_ will exert an inhibitory effect as H_2_O_2_ molecules will consume OH˙ and form other radicals such as HO_2_˙ which slow down the adsorption process.^106^

Fig. 7 shows that increase in adsorbent dosage from 1 g L^−1^ to 5 g L^−1^ at the acidic condition and 30 minutes reaction time lead to an increase in the colour removal efficiency from 60% to 90% and COD removal efficiency from 60% to 73%. As seen in Fig. 7(a) the best colour removal (>90%) was achieved at the dosage of adsorbent between 4–5 g L^−1^ and H_2_O_2_ between 40 mM to 200 mM.

#### (d) Effect of reaction time

**Fig. 8:**
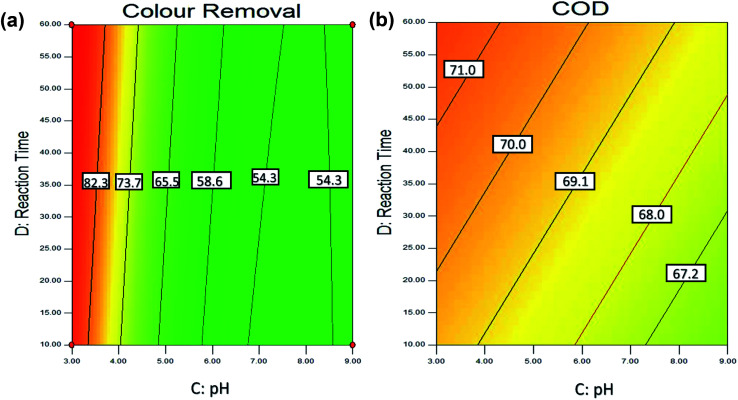
Contour (2D) plot for (a) colour (b) COD removal of reaction time and pH.

Fig. 8(a) and (b) shows the effect of reaction time and pH on the colour removal and COD removal of POME. As discussed earlier, the statistical analysis shows that the reaction time is not a significant parameter, as adsorption process often occurs in the beginning of the reaction. This can be explained as in the beginning stage; the adsorption was rapid as there are many active sites on the adsorbent surface. And, with the passage of contact time, the number of actives sites decreased and more difficult to be penetrated by the pollutants as this is due to repulsive forces between the solute molecules in the solid and the bulk liquid phase.^107^ Besides, adsorbent with a larger surface area required shorter reaction time to achieve the desire adsorption.

Fig. 8(a) and (b) shows that the highest colour removal (>80%) and COD removal (>70%) were obtained within 10 min and 60 min at the acidic condition with 5 g L^−1^ of adsorbent dosage. Previous studies also reported that the adsorption process usually occurs within 15–30 min reaction time^71,72,74^

## 4 Conclusions

This study investigated the possible utilization of palm kernel shell (PKS) for the development of biomass adsorbent. Two types of bio-adsorbent were developed, incorporating PKS with iron oxide and zeolite. The surfaces area, chemical composition, magnetic properties, crystallinity, and stability of the adsorbents were analysed. The adsorption efficiency of newly develop adsorbents was then investigated by using biologically treated POME with the aids of oxidants. Among all the parameters, solution pH was observed to be the most significant parameter affecting the performance of the adsorbent compared to adsorbent dosage, the dosage of H_2_O_2_ and reaction time. Both adsorbents show an excellent efficiency on colour removal (>80%) and COD removal (>60%) under the optimum condition which able to meet the standard set by Department of Environment Malaysia (DOE). The experimental data were found to best fit with Langmuir isotherm model compared to Freundlich and Temkin models. Besides, the adsorption kinetic data follow the pseudo-second-order equation for both adsorbents, which reveals that the adsorption of zeolite-Fe/AC and Fe/AC is monolayer adsorption. This study has proven that palm kernel shell able to convert into a low-cost adsorbent for the adsorption of POME in tertiary treatment. This study could provide a dual benefit to the palm oil industry, as its solid waste can be converted into a useful adsorbent and cost savings in wastewater treatment.

## List of nomenclature and symbol

HO˙: Hydroxyl radical
Fe^2+^: Ferrous ion
*k*
: Reaction rate constant zero order (M/s), first order (1/s), second order (1/Ms)
mM: Molar (mol L^−1^)
min: Minute
s: Seconds
*T*
: Temperature (°C)
*t*
: Time (min)
g L^−1^: Gram per liter
+COD^−^: Chemical oxygen demand
–NH_4^+^_: Ammonium ions
*K*_L_
: Constant of Langmuir isotherm
*K*_f_
: Constant of Freundlich isotherm
*K*_T_
: Constant of Temkin isotherm

## Conflicts of interest

There are no conflicts to declare.

## Supplementary Material

RA-010-D0RA03307C-s001
