# Supplementary material for: Treatment of oil refinery effluent using bio-adsorbent developed from activated palm kernel shell and zeolite
Source: RSC Adv. 2020 Jun 24;10(40):24079–94. doi: 10.1039/d0ra03307c (PMC9055107; doi:10.1039/d0ra03307c)
Supplement: RA-010-D0RA03307C-s001 [file RA-010-D0RA03307C-s001.pdf]

Electronic Supplementary Material (ESI) for RSC Advances.
This journal is © The Royal Society of Chemistry 2020

Supplementary Information

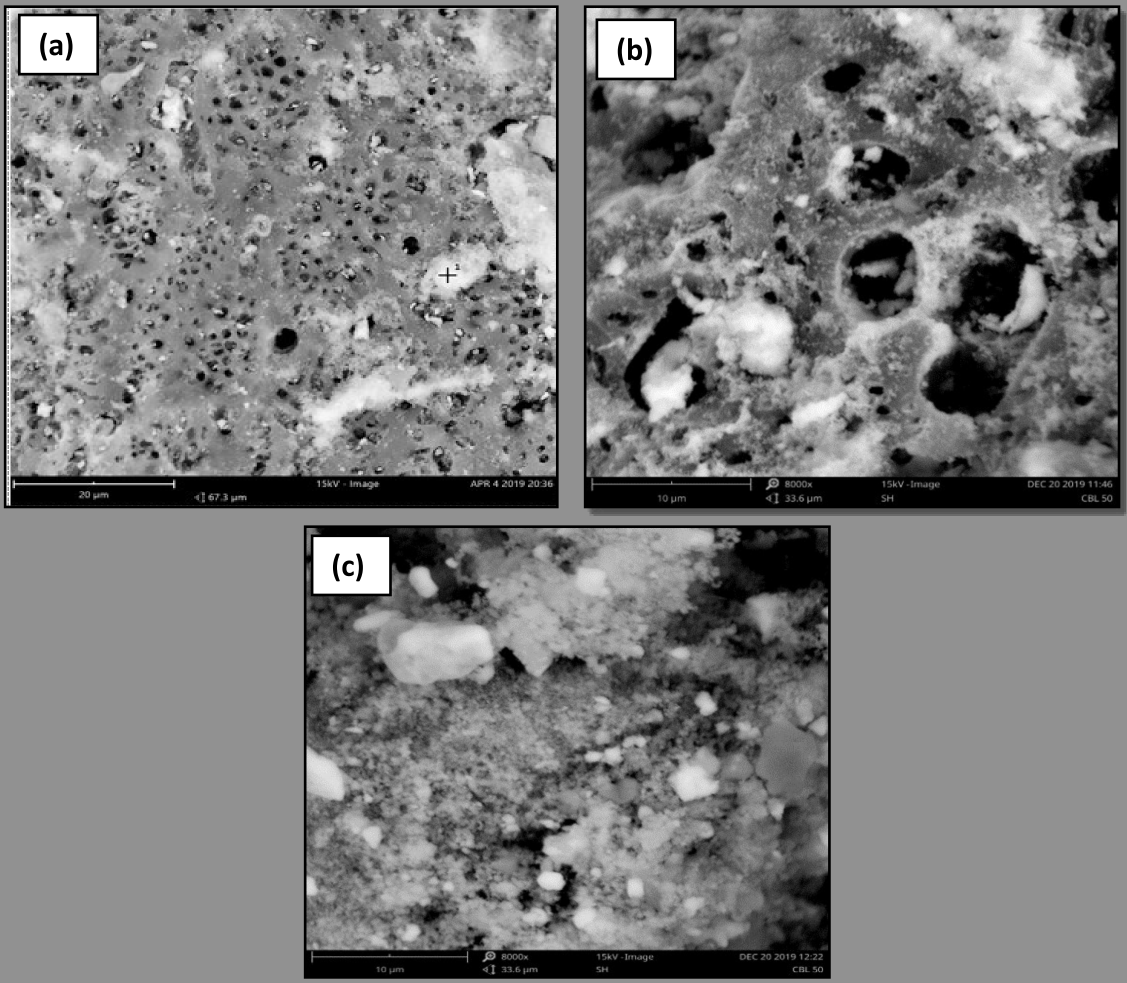

S1: Morphology of (a) AC, (b) Fe/AC and (c) Zeolite-Fe/AC

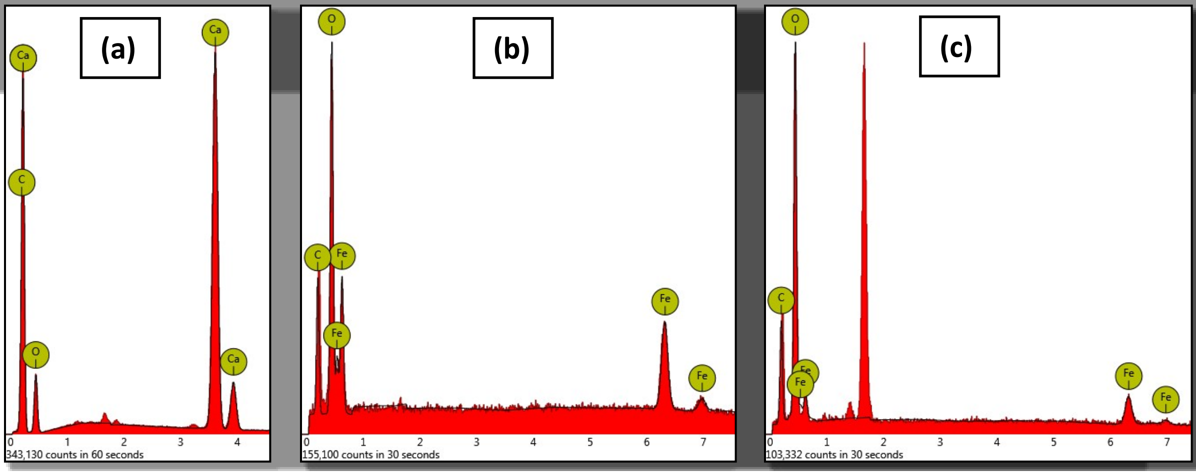

S2: EDX Analysis of (a) AC, (b) Fe/AC and (c) Zeolite-Fe/AC

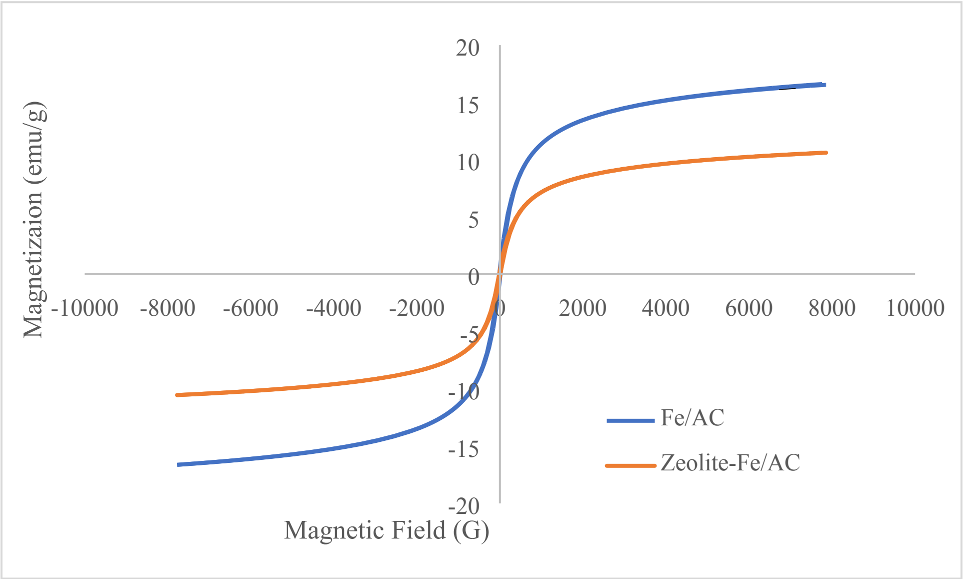

S3: Magnetization curves of Fe/AC and Zeolite-Fe/AC adsorbents

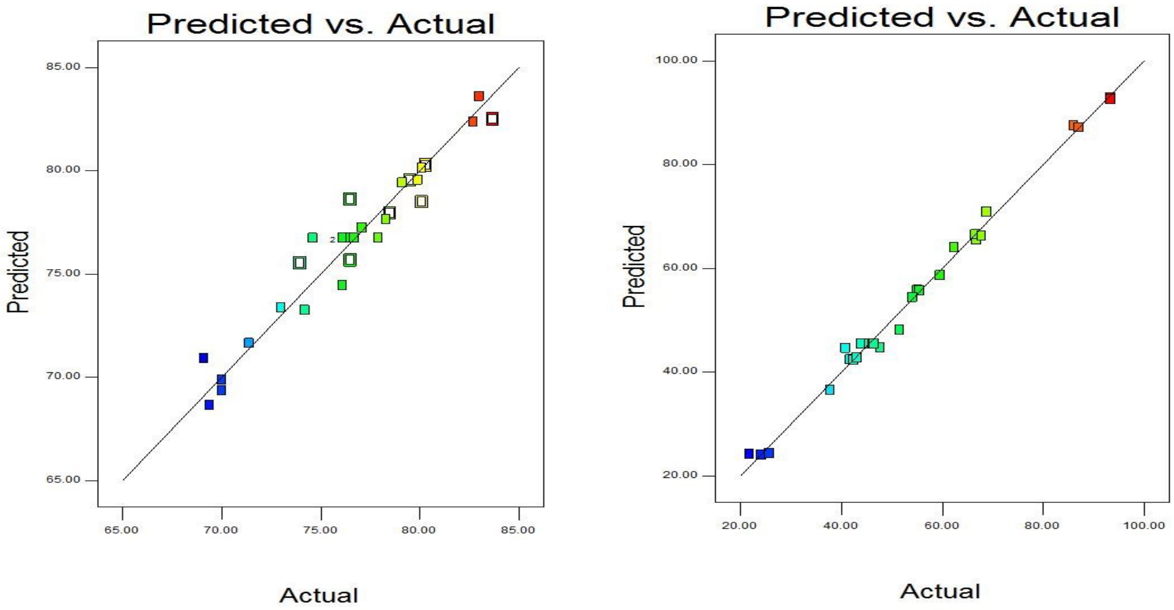

S4: Predicted versus Actual plot for Colour removal **(a)** Fe/AC **(b)** Zeolite-Fe/AC

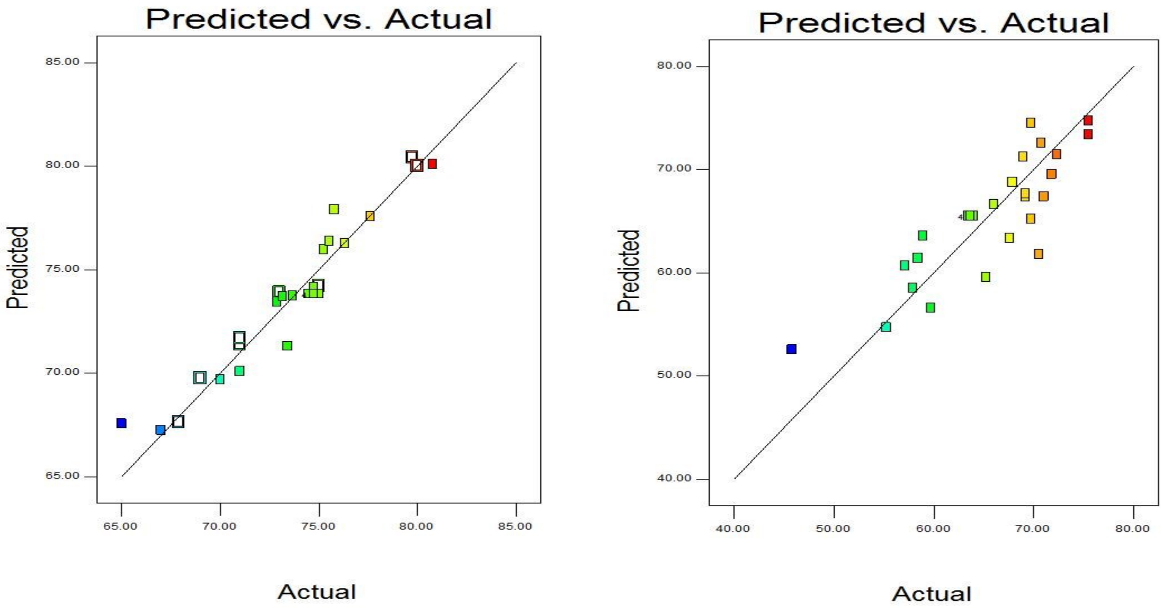

S5: Predicted versus Actual plot for COD removal **(a)** Fe/AC **(b)** Zeolite-Fe/AC

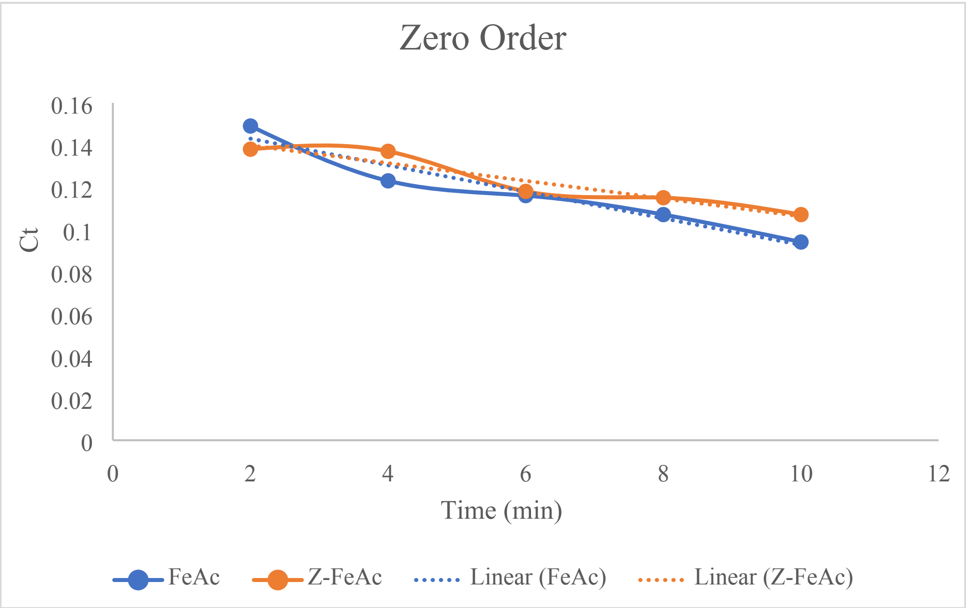

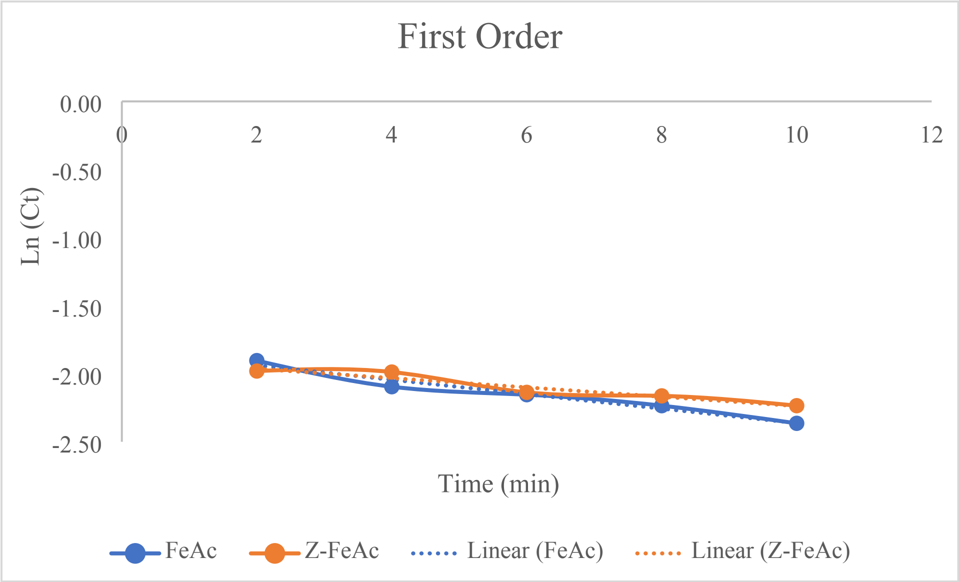

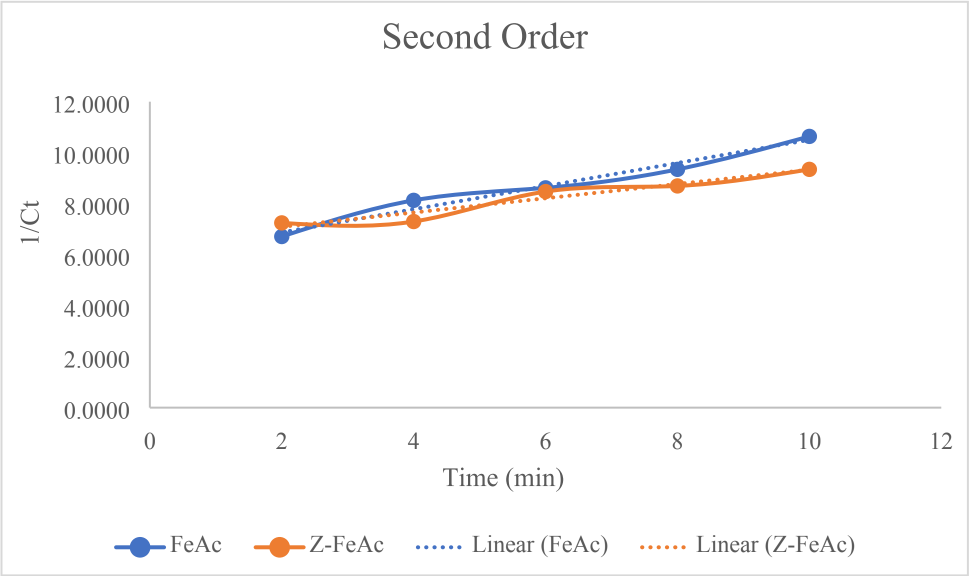

S6: (a) Zero-order, **(b)** First order and **(c)** Second-order reaction kinetic

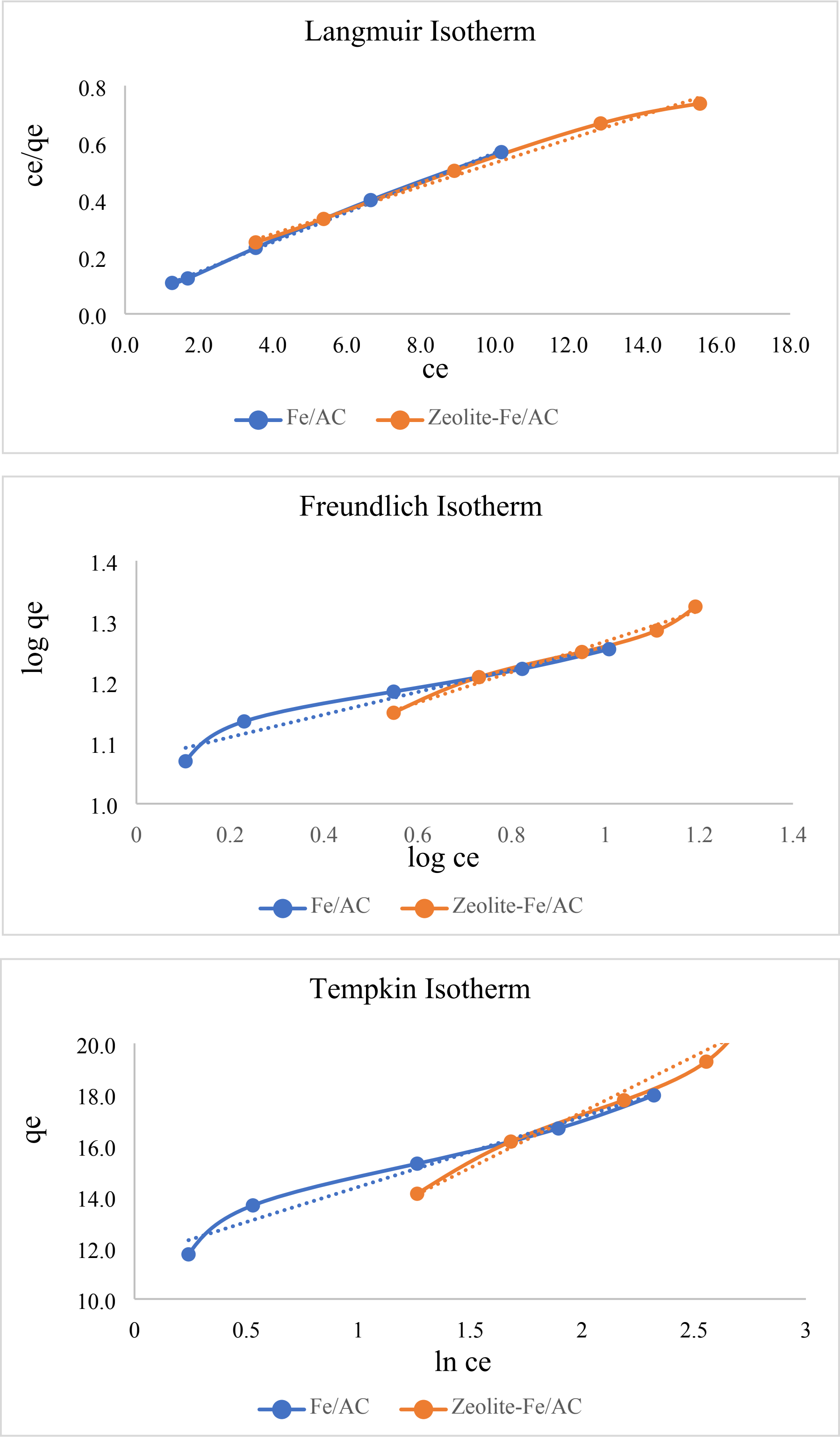

S7: Isotherm model of Fe/AC and Zeolite-Fe/AC **(a)** Langmuir **(b)** Freundlich **(c)** Temkin

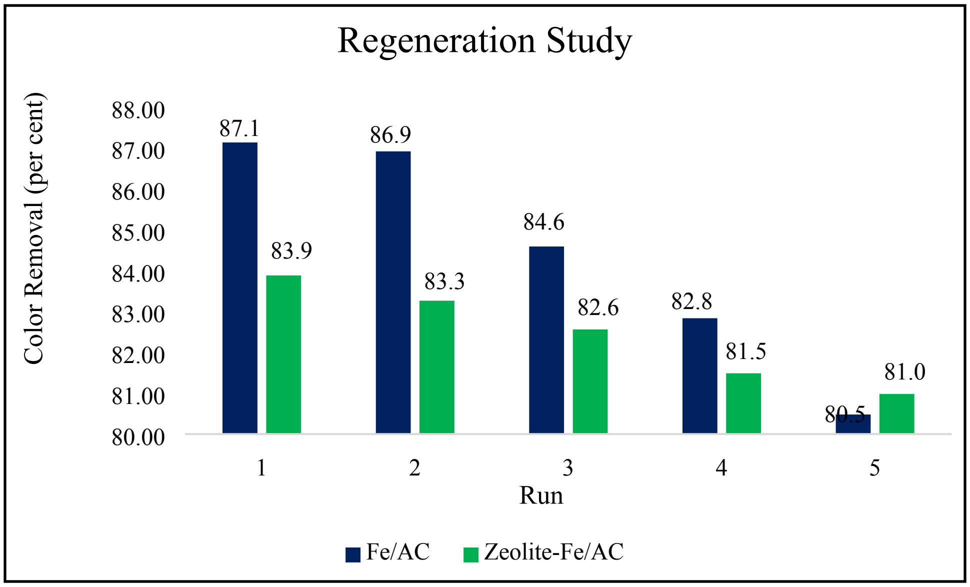

S8: Reusability of adsorbents
